# Impact of post-exertional malaise frequency and fatigue in Long COVID patients on health-related quality of life

**DOI:** 10.1186/s12955-026-02523-x

**Published:** 2026-05-11

**Authors:** Theresa Thölking, Frank Müller, Tim Riester, Viktoria Lampe, Lena-Marie Theil, Eva Hummers, Kousha Sarpari, Alexandra Dopfer-Jablonka, Christine Happle, Sandra Steffens, Mareike Meier-Maiwald, Marie Mikuteit, Dominik Schröder

**Affiliations:** 1 Department of General Practice, University Medical Center, Göttingen, Germany; 2 Department of Family Medicine, Michigan State University, Grand Rapids, MI USA; 3 Department of Rheumatology and Immunology, Hannover Medical School, Hannover, Germany; 4 German Center for Infection Research (DZIF), partner site Hannover- Braunschweig, Hannover, Germany; 5 Department of Pediatric Pneumology, Allergology and Neonatology, Hannover Medical School, Hannover, Germany; 6 German Center for Lung Research, Biomedical Research in End-stage and Obstructive Lung Disease Hannover (BREATH), Hannover, Deutschland; 7 Cluster of excellence RESIST (Resolving infection susceptibility), Hannover, Deutschland; 8 Department of Medical Education, Hannover Medical School, Hannover, Germany; 9 Department of Dermatology and Allergy, Hannover Medical School, Hannover, Germany

**Keywords:** PCS, COVID-19, HRQoL, Quality of life, Post-exertional malaise, PEM

## Abstract

**Purpose:**

The aim of this study was to investigate the impact of post-exertional malaise (PEM) frequency and PEM severity on health-related quality of life (HRQoL) among individuals with Long COVID.

**Methods:**

We conducted a cross-sectional online survey including adults in Germany with self-reported Long COVID and PEM. Fatigue severity was assessed with the Fatigue Assessment Scale (FAS), and HRQoL was measured using the EQ-5D-3L (descriptive index and visual analogue scale [EQ-VAS]). Associations between PEM frequency, fatigue, and HRQoL were examined using correlations and non-parametric group comparisons. Multiple linear regression models were fitted to predict HRQoL while controlling for age, sex, employment status, and subjective social status.

**Results:**

Higher PEM frequency was associated with significantly lower EQ-5D index scores (ρ = − 0.32, *p*<.001). PEM severity was also strongly correlated with reduced HRQoL (EQ-5D index: ρ = − 0.43, *p*<.001). In multivariable regression models, greater fatigue and higher PEM frequency independently predicted poorer HRQoL, even after adjustment for sociodemographic factors.

**Conclusion:**

Both PEM frequency and PEM severity substantially impair HRQoL in individuals with Long COVID. These findings underscore the clinical relevance of PEM as a key symptom and highlight the need for targeted management strategies to mitigate its impact on daily life.

**Clinical trial number:**

German Clinical Trials Register DRKS00026007; registration date: 9 September 2021.

**Supplementary Information:**

The online version contains supplementary material available at 10.1186/s12955-026-02523-x.

## Background

Long COVID refers to a range of health problems that continue or newly appear after an acute COVID-19 infection. There is no uniform definition of Long COVID, and the criteria used by different institutions vary. The World Health Organization (WHO) defines it as symptoms that typically emerge three months after acute SARS-CoV-2 infection, persist for at least two months, and cannot be explained by an alternative diagnosis [1]. The Centers for Disease Control and Prevention (CDC) defines Long COVID as persisting symptoms for at least three months [2]. Commonly reported manifestations include – besides many other possible symptoms – fatigue, cognitive dysfunction, and post-exertional malaise or symptom exacerbation (PEM/PESE), which is characterized by a delayed worsening of symptoms following physical or mental activity, often lasting for several days or longer [2, 3]. Recent research indicates that PEM, sometimes described as “PEM-crashes,” may occur when physical or cognitive exertion exceeds an individual threshold [4]. In severe cases, individuals can become significantly debilitated and even dependent on care or daily assistance [5].

Although no causal cure currently exists, several symptom-oriented treatment approaches have been developed and incorporated into clinical guidelines, such as the German S1 guideline for Long/Post-COVID [6]. Nevertheless, despite the existence of such guidelines, patients frequently report substantial barriers in accessing adequate care, including a lack of recognition, limited specialist availability, and long waiting times [5, 7].

Long COVID is associated with substantial societal and individual consequences. Notably, many affected individuals are relatively young and of working age, highlighting the considerable socioeconomic burden associated with Long COVID [8]. Studies have demonstrated that Long COVID is linked to reduced work ability and occupational changes, including reduced working hours and job loss, underscoring its profound impact on productivity and employment structures [5, 9].

Affected people also frequently report significant and persistent reductions in health-related quality of life (HRQoL) and restrictions in daily functioning [10, 11]. Multiple studies have found significantly lower HRQoL among individuals with Long COVID compared with population norms or control groups, with impairments persisting months after the initial infection [12–14]. The 2024 National Academies of Sciences, Engineering, and Medicine report stresses that PEM in Long COVID is strongly associated with factors negatively impacting HrQoL, including greater risks of cognitive impairment (“brain fog”), sleep disturbance, pain, and more severe functional and occupational limitations compared to Long COVID without PEM [15]. These findings highlight the relevance of PEM as a potential key driver of impaired HRQoL, underscoring its importance for both research and clinical management.

Against this background, the present study aims to examine how both frequency and self-perceived severity of PEM affect HRQoL in individuals with Long COVID. By distinguishing between how often PEM episodes occur and how strongly they are experienced, the study seeks to provide a more differentiated understanding of their respective contributions to impairments in daily functioning and overall well-being. Such findings are essential for identifying the mechanisms through which PEM leads to a reduced HRQoL and to guiding the development of patient-centered treatment strategies.

## Methods

### Study design and participants

This study is part of the multi-center, interdisciplinary longitudinal DEFEAT Corona project investigating the health and social consequences of the COVID-19 pandemic. The present work reports a secondary cross-sectional analysis based on data from the fourth survey wave.

Data were collected via an online survey administered through the SoSci Survey platform (SoSci Survey GmbH, Munich, Germany). The survey, available exclusively in German, was launched in September 2021 through the project website (defeat-corona.de). Access was promoted using a QR code placed on posters and flyers distributed in public spaces (e.g., libraries, universities, sports clubs, churches, and town halls) as well as in medical and research institutions, primarily across Lower Saxony, Germany. In addition, information leaflets were mailed to 400 randomly selected primary care, internal medicine, radiology, and occupational therapy practices within the region. To increase outreach, patient advocacy groups were invited to share the survey link on social media platforms such as Facebook, and the project was also featured on a national Long COVID website (longcoviddeutschland.org). The study was promoted as a digital Long COVID project about the long-term consequences of the COVID-19 pandemic not specific regarding PEM.

Eligible participants were adults (≥ 18 years old) residing in Germany regardless of SARS-CoV-2 infection or Long-COVID status. Long COVID was defined as the presence of new or persisting symptoms four weeks or more after an acute SARS-CoV-2 infection, confirmed through a PCR test, antigen test, or antibody test, based on self-reported information. Prior to participation, individuals provided digital informed consent and confirmed their age. No personally identifying information was collected; instead, pseudonyms were assigned to enable follow-up. The study team remained available to participants via email, phone, or WhatsApp for additional inquiries. Detailed methodology is described in the study protocol [16]. This analysis is based on a follow-up survey conducted from March 2025 until April 2025.

Ethical approval was obtained from the institutional review boards of Hannover Medical School (9948_BO_K_2021) and the University Medical Center Göttingen (29/3/21). The study is registered in the German Clinical Trials Register (DRKS00026007). Results are reported following the STROBE guidelines for cross-sectional studies [17].

## Measures

### EQ-5D-3L

The EQ-5D-3L consists of two components: the descriptive system (EQ-5D index) and a visual analogue scale (VAS) [18]. The descriptive system covers five domains of health: mobility, self-care, usual activities, pain/discomfort, and anxiety/depression, each with three response options: no problems, some problems, and extreme problems. Responses across the five domains form a five-digit health state profile, which can be converted into an index score ranging from 0 (indicating worst health) to 1 (indicating best health), representing overall HRQoL [19]. For descriptive and regression analyses, the EQ-5D index was multiplied by 100 and reported on a 0–100 scale to improve interpretability and to align the scale with the EQ-5D VAS (higher values indicate better HRQoL). In this study, EQ-5D-3L health states were converted to index values using the German population-based time trade-off (TTO) value set [19]. The VAS provides a subjective rating of current health on a scale from 0 to 100, anchored at 0 = “worst imaginable health state” and 100 = “best imaginable health state.”

The EQ-5D-3L is one of the most widely used standardized instruments for assessing HRQoL in both general populations and clinical samples. It has been applied across a broad range of conditions, including chronic fatigue, pain, and other long-term health impairments, and has demonstrated good reliability and sensitivity to differences in health status [20, 21]. Its brevity and standardized scoring system make it particularly suitable for use in large-scale surveys and epidemiological research [22].

### Fatigue measures

#### PEM frequency

The frequency of post-exertional malaise (PEM) episodes was assessed using an open-ended text field, as no well-established instruments exist to reliably capture PEM frequency. Prior to this question, PEM was defined in the questionnaire as “exertion-induced symptom worsening after mild activity”. Participants were then asked: “How often do you experience PEM?” Responses were subsequently classified manually by two independent coders (T.R. & V.L.) into four categories reflecting occurrence frequency: daily, weekly, monthly, and less frequent. The category ‘less frequent’ denotes PEM occurring less often than monthly (e.g., ‘every 2–3 months’, ‘a few times per year’). Classification was based on stringent criteria, with participants’ responses required to provide explicit frequency information that could be unambiguously allocated to one of the four categories (see S1 for the detailed classification scheme). Responses lacking clarity or containing ambiguity were deemed unassignable.

#### Fatigue assessment scale

The Fatigue Assessment Scale (FAS) is a 10-item self-report questionnaire designed to evaluate chronic fatigue symptoms, covering both physical and mental aspects [23]. We used a German translation of the FAS, a version that has been applied in prior research, demonstrating its feasibility and reliability in German-speaking populations [24, 25]. Respondents select from five response categories ranging from never (0) to always (4). Items 1–3 and 5–9 are scored directly (0 to 4), whereas items 4 and 10 are inverse-scored (i.e. 0 ↔ 4) to correct for phrasing. The total score is computed by summing all item responses, yielding a range from 0 to 40, with higher values indicating greater fatigue severity [26]. Internal consistency has been shown to be high, with Cronbach’s α values around 0.90 across different populations [23, 27]. Test–retest reliability has also been reported as satisfactory (*r* =.89 over one week) [27]. Construct validity is supported by significant correlations with related measures, such as the vitality subscale of the SF-36, and by the ability of the FAS to distinguish between clinical and non-clinical groups [23, 27]. In our study, fatigue severity (FAS) was used as a measure for PEM severity, because previous research demonstrates substantial overlap and use of the FAS in Long-Covid/post-COVID contexts where PEM is a key symptom. Thus, the FAS score was considered an acceptable indicator of PEM severity in this context [23, 26].

### Covariates

We included self-reported age, gender, employment status, number of comorbidities and subjective social status as covariates in the analysis. Age was treated as a continuous variable (in years). Participants reported their gender in three categories (female, male, non-binary) and their employment status in four categories (full-time, part-time (at least 15 h a week), marginal/irregular part-time (less than 15 h a week), not employed). Comorbidities were assessed using a self-reported checklist comprising 34 predefined medical conditions. Each condition was recorded as a dichotomous variable (yes/no). The list included cardiovascular, metabolic, pulmonary, autoimmune, neurological, psychiatric, and other chronic conditions, as well as an open category for “other diseases (see S2 for a full list).

For the present analysis, a summary variable (“number of comorbidities”) was calculated by summing all selected conditions across the 34 items, resulting in a continuous count variable reflecting the total burden of self-reported comorbidities per participant.

Subjective social status was assessed using the MacArthur Scale of Subjective Social Status [28], where participants rated their perceived position in society on a 10-rung ladder, with higher rungs indicating higher social standing. This scale captures individuals’ perception of their own social standing relative to others, reflecting not only income or education but also psychosocial aspects such as respect, influence, and life opportunities. Subjective social status was included as a covariate because perceived social standing has been shown to influence health outcomes and quality of life independently of objective socioeconomic indicators [29].

### Statistical analysis

Participants who reported having neither Long COVID nor PEM were excluded for statistical analyses.Descriptive statistics were calculated for sociodemographic variables (sex, age, employment status), reporting categorical variables as absolute and relative frequencies and continuous variables as mean with standard deviation (SD). In addition, responses for each EQ-5D-3L dimension (mobility, self-care, usual activities, pain/discomfort, and anxiety/depression) were summarized descriptively by reporting the distribution of severity levels (levels 1–3) for each domain. We also calculated the proportion of participants reporting any problems (levels 2–3) in each EQ-5D dimension stratified by PEM frequency. Descriptive statistics are based on the full sample (*n* = 161). Because this is a secondary analysis of existing data, no a priori power analysis was performed. The EQ-5D index (0–1) was derived using the German EQ-5D-3L value set [30]. To ensure comparability with the VAS, the original 0–1 index scores were multiplied by 100, resulting in a 0–100 scale. We examined associations between PEM frequency, fatigue, and HRQoL using Spearman’s correlations and non-parametric group comparisons (Kruskal–Wallis tests with pairwise Wilcoxon tests, adjusted for multiple comparisons using the Benjamini–Hochberg procedure). We fitted multiple linear regression models to predict HRQoL (EQ-5D index and EQ-VAS) from PEM frequency and fatigue, adjusting for age, sex (female vs. male), employment status, number of comorbidities and subjective social status. Only complete cases were included in the regression analyses. Because only one participant selected the non-binary option, regression analyses were restricted to participants reporting female or male (regression sample *n* = 160). For regression analyses, employment status was recoded by collapsing marginal/irregular part-time (< 15 h/week) into the part-time category. Model assumptions were checked with standard diagnostic plots. To assess potential non-linearity between fatigue severity and the EQ-5D, we additionally fitted a generalized additive model (GAM) with a penalized spline term for fatigue severity (REML; k = 5), using the same covariates as in the primary model. As the smooth term indicated no evidence of non-linearity, fatigue severity was modelled as a linear term in the final regression analyses. Regression results are reported as regression coefficients with 95% confidence intervals and p-values, along with the overall model fit (R²). We considered p-values < 0.05 (two-tailed) statistically significant. All analyses and plots were conducted in R (version 4.4.2), using the packages dplyr [31], tidyr [32], ggplot2 [33], ggpubr [34], eq5d [30], sjPlot [35], kableExtra [36], knitr [37], magrittr [38], and rstatix [39].

## Results

### Participant characteristics

**Fig. 1:**
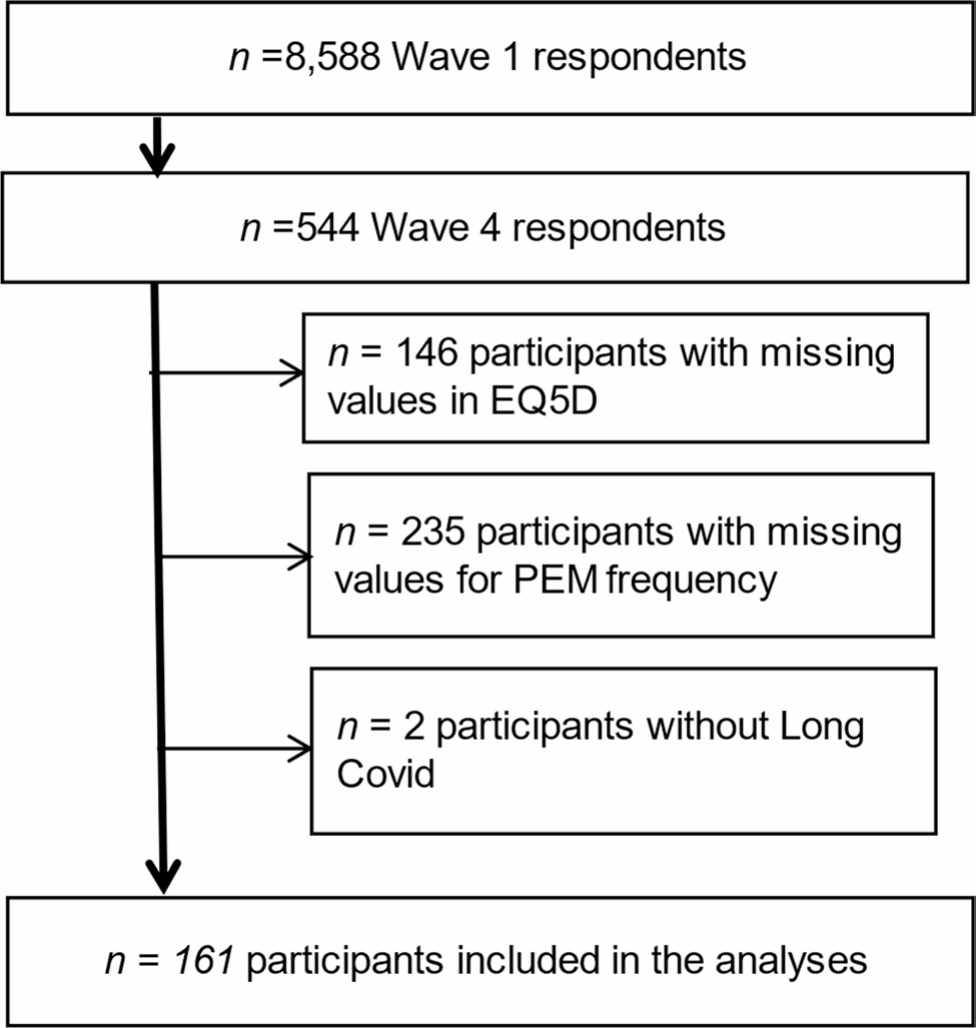
Flowchart of included participants

After applying the exclusion criteria, 161 participants were included in the analysis (Fig. 1). The analysis is based on participants who responded to the fourth survey wave of the cohort (*n* = 544), derived from an original cohort of 8,588 participants enrolled in the first wave. Participants were excluded due to missing EQ-5D data (*n* = 146), missing information on PEM frequency (*n* = 235), or because they did not report Long COVID (*n* = 2).

Participants had a mean age of 45.89 years (SD = 10.94). The majority were female, and most were not employed. More than half of the sample (52.2%) reported experiencing PEMs on a weekly basis. All participant characteristics are presented in Table 1. Mean and median of fatigue severity and PEM frequency are presented in Table S3.

**Table 1:** Participant characteristics of the study sample

Characteristic	Descriptive
**Gender**, n (%)	
Diverse	1 (0.6%)
Female	137 (85.1%)
Male	23 (14.3%)
**Age** (mean [SD])	45.89 (10.94)
20–34 years, n (%)	28 (17.4%)
35–49 years, n (%)	68 (42.2%)
50–64 years, n (%)	65 (40.4%)
**Year of Last SARS-CoV-2 Infection**, n (%)	
missings	4 (2.5%)
2019	1 (0.6%)
2020	4 (2.5%)
2021	7 (4.4%)
2022	50 (31.1%)
2023	48 (29.8%)
2024	42 (26.1%)
2025	5 (3.1%)
**Employment status** n (%)	
Employed full-time,	22 (13.7%)
Employed part-time	34 (21.1%)
Marginal/irregular part-time	8 (5.0%)
Not employed	97 (60.2%)
**MacArthur Scale** (mean [SD])	4.8 (1.87)
1–3, n (%)	34 (22.1%)
4–7, n (%)	114 (74.0%)
8–10, n (%)	6 (3.9%)
**PEM Frequency** n (%)	
Daily	19 (11.8%)
Weekly	84 (52.2%)
Monthly	53 (32.9%)
Less	5 (3.1%)

SD: standard deviation

### PEM frequency and health-related quality of life

**Fig. 2:**
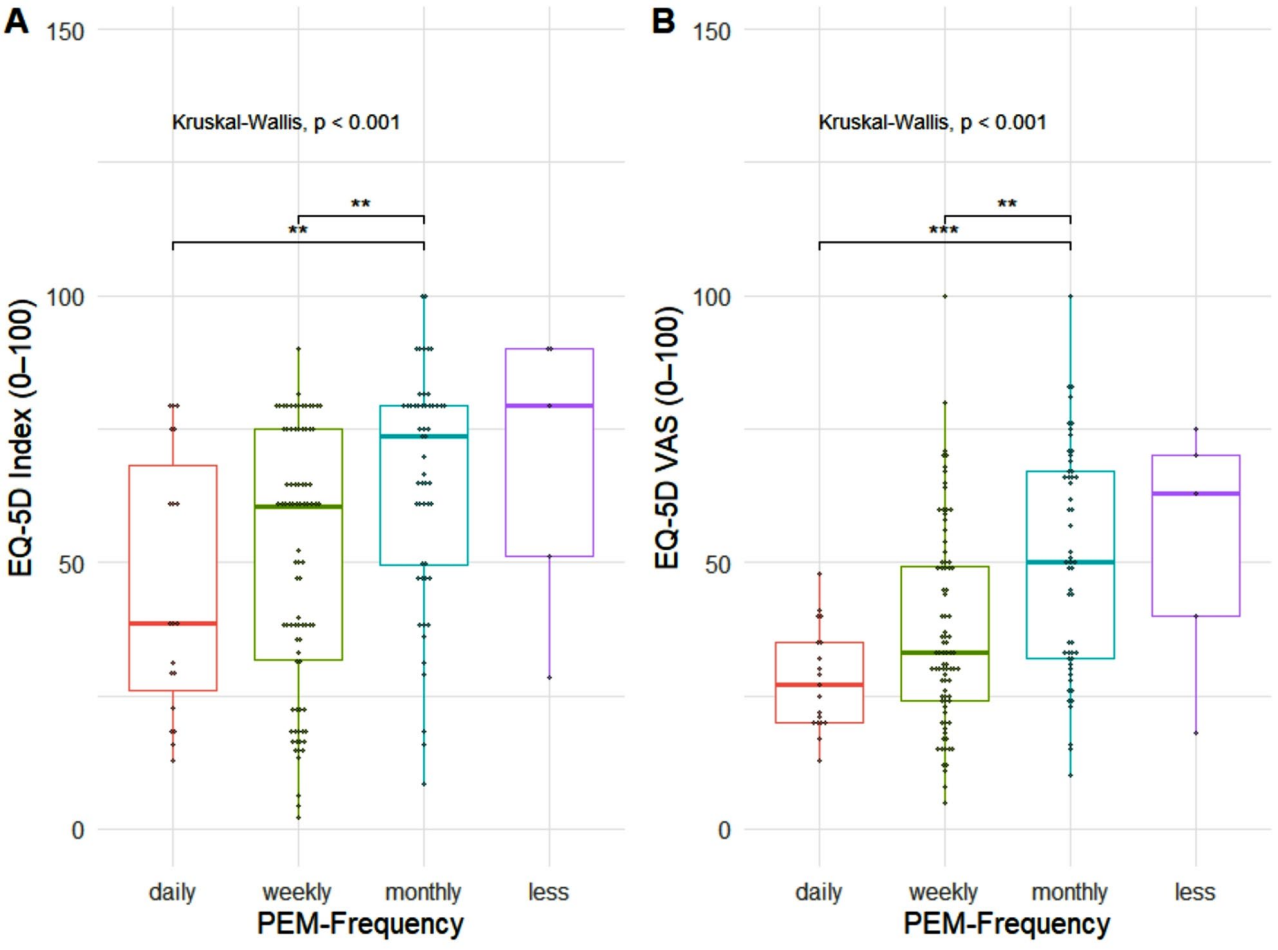
Boxplots of EQ-5D-3L index and EQ-5D VAS stratified after PEM-Frequency (*n* = 161). Note: P-values in the figure are shown in scientific notation where applicable

The mean HRQoL, as measured by the EQ-5D index, was 55.26 (SD = 24.66), whereas the mean EQ-VAS score was lower, at 40.91 (SD = 20.26). PEM frequency was significantly associated with both the EQ-5D index and EQ-VAS (Fig. 2). Significant group differences could be observed across PEM frequency categories (all *p* <.01). Pairwise comparisons revealed that participants with daily PEM reported the lowest levels of HRQoL, whereas those experiencing PEM less than monthly had the highest scores. In line with this, Spearman correlations confirmed significant negative associations between PEM frequency and the EQ-5D index (ρ = − 0.32, *p* <.001) as well as the EQ-VAS (ρ = − 0.37, *p* <.001).

### Distribution of EQ-5D-3L responses across domains

To further characterize health-related quality of life in the study population, we examined the distribution of responses across the five EQ-5D-3L dimensions (Table 2). Problems were most frequently reported in the domains of pain/discomfort and usual activities. In the pain/discomfort dimension, 93.2% of participants reported at least some problems, including 76.4% with moderate problems (level 2) and 16.8% with severe problems (level 3). Similarly, limitations in usual activities were common, with 91.9% of participants reporting impairment (56.5% level 2 and 35.4% level 3). Mobility problems were also frequent, affecting 59.0% of participants overall.

In contrast, impairments in self-care were less common, with 29.2% of participants reporting any difficulties. Anxiety/depression problems were reported by 33.5% of participants, the majority of which were moderate. Overall, these findings indicate that pain/discomfort and limitations in usual activities represent the most prominent contributors to reduced health-related quality of life in this cohort. To complement these overall domain-level distributions, the proportion of participants reporting any problems (levels 2–3) in each EQ-5D-3L dimension stratified by PEM frequency is provided in Table S5.

**Table 2:** Distribution of EQ-5D-3L responses by dimension and severity level

Level/Domain	Mobility	Self-care	Usual activities	Pain/discomfort	Anxiety/depression
Level 1	66 (41.0)	114 (70.8)	13 (8.1)	11 (6.8)	107 (66.5)
Level 2	85 (52.8)	45 (28.0)	91 (56.5)	123 (76.4)	48 (29.8)
Level 3	10 (6.2)	2 (1.2)	57 (35.4)	27 (16.8)	6 (3.7)
Total	161 (100)	161 (100)	161 (100)	161 (100)	161 (100)

Values are presented as n (%). Percentages represent the proportion of participants within each EQ-5D dimension reporting a given severity level. Level 1 indicates no problems, level 2 moderate problems, and level 3 severe problems

### Fatigue severity and health-related quality of life

**Fig. 3:**
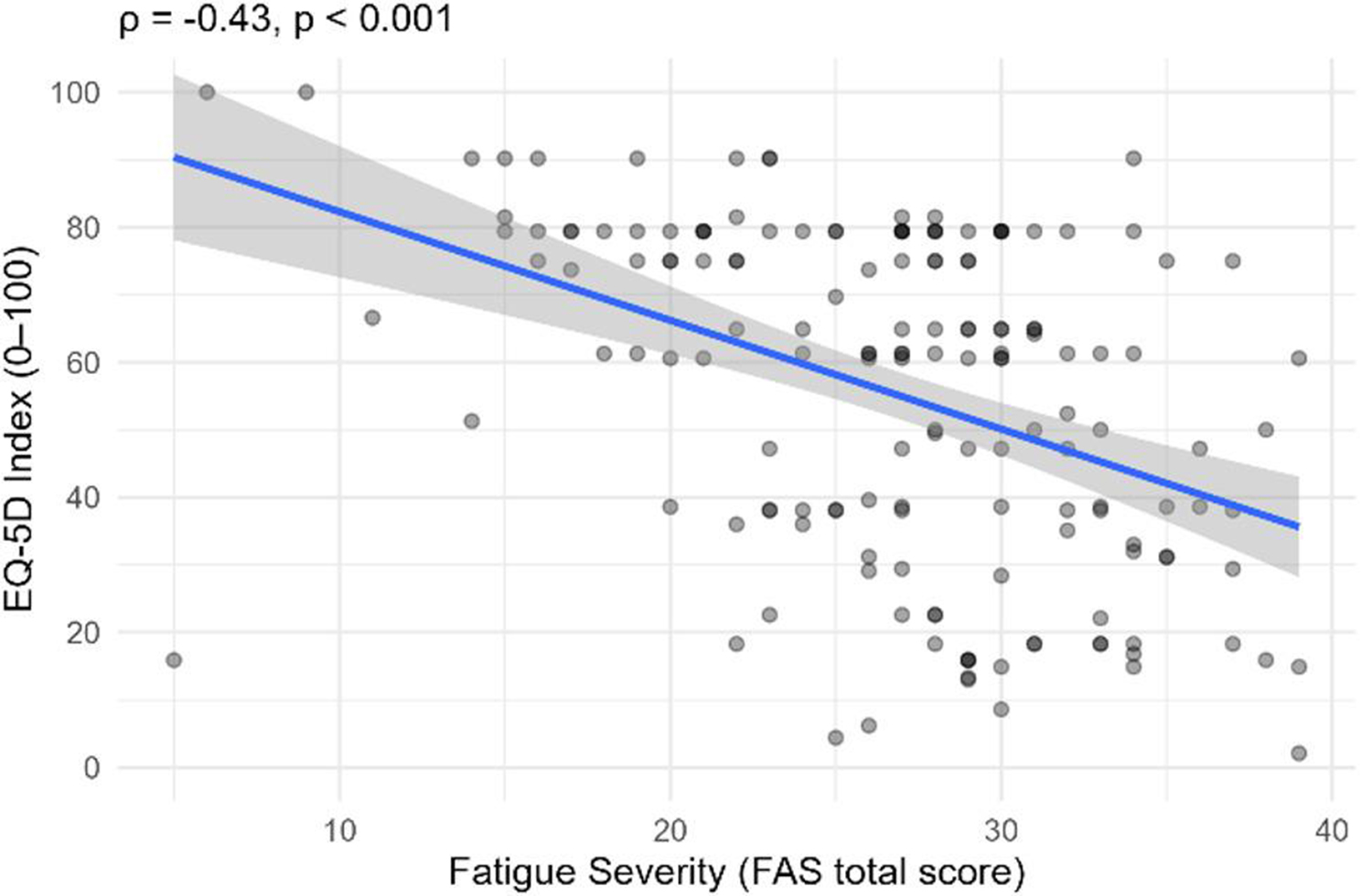
Scatterplot showing the association between fatigue severity (FAS total score) and HRQoL (EQ-5D-3L index, 0–100). The line shows the fitted linear regression; the shaded band indicates the 95% confidence interval for the mean estimate. R² = 0.18

**Fig. 4:**
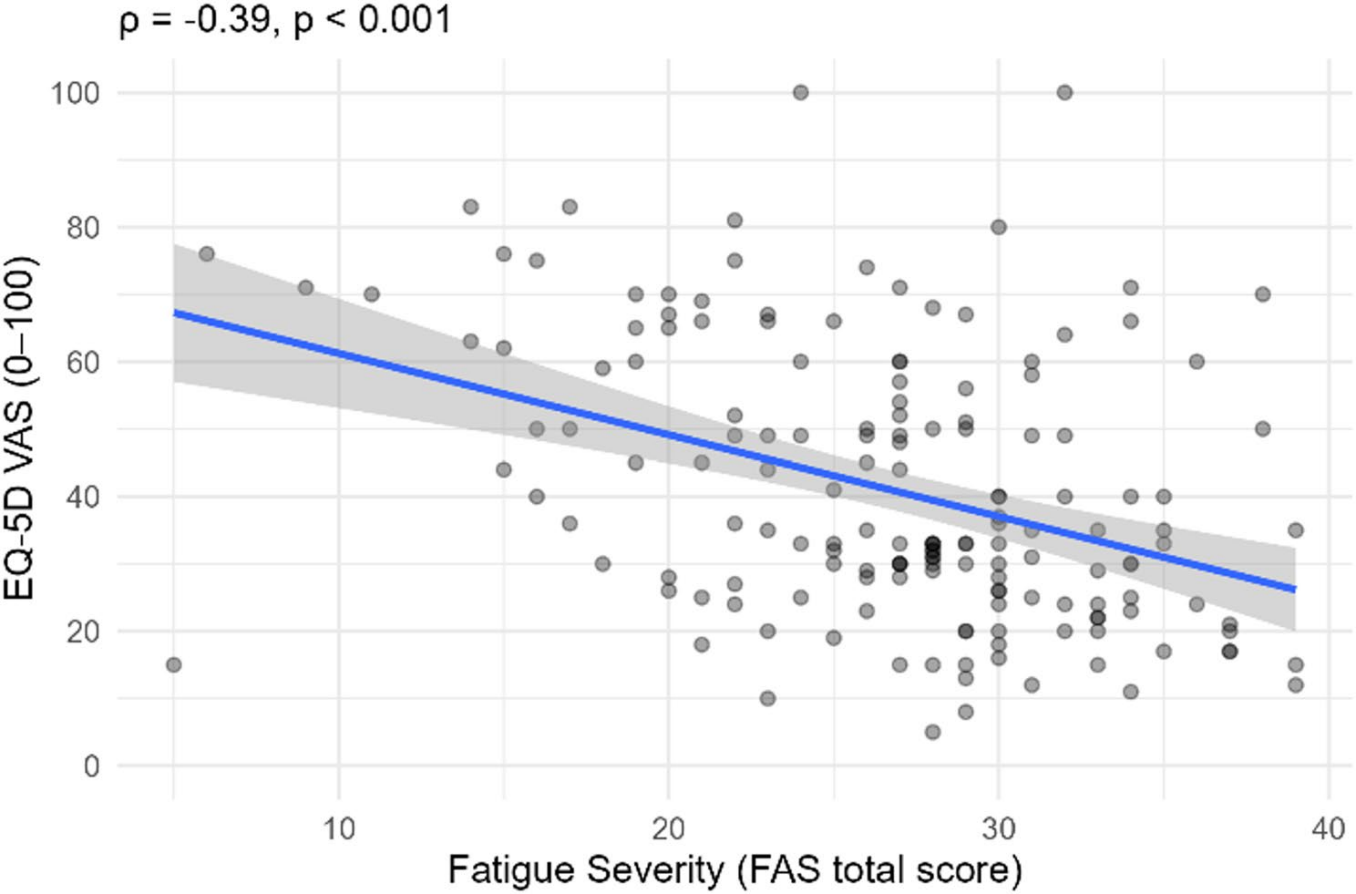
Scatterplot showing the association between fatigue severity (FAS total score) and HRQoL (EQ-VAS, 0–100). The line shows the fitted linear regression; the shaded band indicates the 95% confidence interval for the mean estimate. R² = 0.15

Fatigue severity, measured with the FAS, showed significant associations with HRQoL. Higher FAS scores were significantly correlated with lower EQ-5D index values (ρ = − 0.43, *p* <.001; see Fig. 3) and lower EQ-VAS scores (ρ = − 0.39, *p* <.001; see Fig. 4).

### Multivariable analysis on health-related quality of life

In the multiple linear regression models, higher fatigue severity was a significant independent predictor of lower HRQoL in the EQ-5D index model (Table 3). Specifically, each one-point increase in FAS score was associated with a decrease of 0.98 points in the EQ-5D index (*p* = .021), while no significant association was observed for EQ-VAS.

For EQ-VAS, PEM frequency showed associations: compared with daily PEM, monthly PEM was significantly associated with better self-rated health (β = 14.84, *p* = .017), whereas weekly PEM showed a borderline effect (β = 10.57, *p* = .051). Unemployed participants reported significantly lower HRQoL in both models, while age was positively associated with the EQ-5D index only. Gender was not a significant predictor. All regression coefficients, along with 95% confidence intervals and p-values, are presented in Table 3.

**Table 3:** Multivariable Regression on health-related Quality of Life, *n* = 160 participants included in analyses

Covariate	Category	EQ-5D Index		EQ-VAS	
		Β (95% CI)	*p*	Β (95% CI)	*p*
Age (in years)		0.48 (0.08–0.89)	**0.022**	0.21 (-0.10–0.52)	0.189
Gender (female vs. male)	Male	-3.27 (-19.06–12.53)	0.686	-9.23 (-21.16–2.70)	0.133
	Female	ref.	-	-	
Employment status	Unemployed	-24.66 (-42.00 – -7.32)	**0.007**	-20.83 (-33.93 – -7.73)	**0.003**
	Part-time	-5.29 (-22.69–12.11)	0.553	-8.58 (-21.73–4.56)	0.204
	Full-time	ref.	-	-	
Mac Arthur Scale		1.13 (-1.97–4.23)	0.478	0.42 (-1.93–2.77)	0.726
FAS (Scale 0–40)^1^		-0.98 (-1.80 – -0.16)	**0.021**	-0.19 (-0.81–0.43)	0.542
PEM Frequency	Daily	ref.	-	-	
	Weekly	4.20 (-9.62–18.02)	0.553	10.57 (0.13–21.01)	0.051
	Monthly	5.13 (-10.68–20.95)	0.526	14.84 (2.90–26.78)	**0.017**
	Less	3.86 (-29.95–37.67)	0.824	11.17 (-14.37–36.70)	0.394
Comorbidities		-0.74 (-2.85–1.36)	0.491	-1.52 (-3.11–0.07)	0.064
		R² = 0.339 adj. R² = 0.255		R² = 0.334 adj. R² = 0.250	

PEM = Post-Exertional Malaise; FAS = Fatigue Assessment Scale; CI = Confidence Interval; ref.: reference category. ^1^ Higher scores indicate greater fatigue severity

## Discussion

This cross-sectional study with 161 participants examined how PEM frequency and severity relate to HRQoL in individuals with Long COVID. Three main findings emerged:

PEM frequency and HRQoL: Higher PEM frequency was associated with lower EQ-5D index and lower EQ-VAS scores. This aligns with recent large-scale evidence showing that individuals reporting Long COVID have significantly worse utility scores on EQ-5D-5L than other chronic diseases [40].

PEM severity and HRQoL: PEM severity was associated with reduced HRQoL even when accounting for PEM frequency. This is consistent with the observation that chronic fatigue is a defining symptom in Long COVID and is associated with quality-of-life impairments [41].

Multivariable findings: In an adjusted regression model, higher PEM frequency was significantly associated with lower EQ-VAS scores, with participants reporting monthly PEM showing an average increase of 14.8 points compared with daily PEM, even after adjusting for age, sex, employment status, subjective social status, fatigue severity, and number of comorbidities. No significant associations were observed between PEM frequency and EQ-5D index scores. Fatigue severity was negatively associated with EQ-5D, but not with EQ-VAS. Employment status also showed a significant impact on HRQoL, with unemployed participants reporting lower scores on both instruments. These findings indicate that frequent PEM may predominantly affect patients’ overall perceived health (EQ-VAS), whereas dimension-specific HRQoL measures (EQ-5D) may be less sensitive to symptom fluctuations, highlighting the importance of assessing both general and specific QoL outcomes in this population.

The findings fit into a growing body of literature on HRQoL in people with Long COVID. For example, Sun, et al. reported significantly lower EQ-5D-3L index values in Long COVID patients compared to non-Long COVID controls [42]. A longitudinal study likewise found reduced HRQoL several years after SARS-CoV-2 infection [43]. Other work has emphasized the prevalence and impact of PEM in Long COVID cohorts [44].

The mean EQ-5D index in our cohort was 55.26 on a 0–100 scale (about 0.55 on the conventional 0–1 utility scale), and the mean EQ-VAS was 40.91, indicating substantial HRQoL impairment. These values are comparable to a cross-sectional sample of individuals with self-reported post-acute sequelae of SARS-CoV-2 infection, which reported a mean EQ-5D-5L utility of 0.51 and mean EQ-VAS of 41.6 [45]. Our EQ-5D index is also close to a post-COVID cohort assessed several months after infection (mean EQ-5D-3L index 0.57), although that cohort reported a higher EQ-VAS (56.6) [46]. In contrast, a large German online survey reported higher HRQoL in its Long-Covid group (mean EQ-5D-3L index 0.66; EQ-VAS 57.6) [14]. Conversely, other population samples with self-reported Long Covid have reported lower mean EQ-5D index values around 0.49 [40]. Differences across cohorts likely reflect heterogeneity in Long Covid definitions, recruitment setting (community vs. clinic vs. online), time since infection, symptom severity mix, and EQ-5D version/value-set choices, which limits direct comparability.

Importantly, the overall HRQoL reduction among participants reporting PEM (mean EQ-5D-3L = 55) is clinically substantial. This value lies in the lower range of utilities reported for chronic conditions and is comparable to EQ-5D-3L means observed in catalogues for disorders such as dementia (54.6) and post-traumatic stress disorder (55.7), as reported in a large Danish population-based EQ-5D-3L catalog [47]. In contrast, pooled estimates for conditions like chronic heart failure fall in the interquartile range of 64–72, indicating generally higher HRQoL [48]. These comparisons indicate that the HRQoL burden associated with PEM/Long COVID can reach levels observed in other serious chronic illnesses.

Our results suggest that while PEM frequency is an important dimension, PEM severity may have a more direct and stable relationship with HRQoL. Clinically, this implies further research and interventions should address both PEM dimensions. Rehabilitation approaches, including individualized, symptom-titrated exercise, have shown promise in reducing fatigue and improving HRQoL in Long COVID populations [49].

### Limitations

This study has several limitations. First, its cross-sectional and online-only design limits causal inference and generalizability. Moreover, only a subset of participants completed all relevant questions on HRQoL, fatigue, and PEM frequency, which may have introduced attrition bias. A comparison of included participants and Long COVID participants with missing data revealed that excluded individuals were, on average, older. No significant differences were observed with respect to the gender distribution and number of comorbidities (see S4). These suggest the presence of potential selection bias, as certain demographic groups (older participants) were underrepresented in the analyzed sample. Importantly, the similarity in comorbidity burden between groups indicates that clinical complexity is unlikely to be a major source of bias. Nevertheless, caution is warranted when generalizing the results to the full population, particularly with regard to age- related outcomes.

In addition, self-selection bias is possible due to voluntary online participation and dissemination via Long Covid–related channels, which may have overrepresented individuals with higher symptom burden and lower HRQoL. The net direction of bias is uncertain because severely affected individuals may also be less likely to complete a survey; therefore, representativeness may be limited and bias in the observed associations cannot be ruled out. All data were self-reported, including COVID-19 status, which may be prone to recall bias or misclassification.

The study sample showed an imbalanced sex (female vs. male) distribution, with a substantially higher proportion of women (137 women vs. 23 men). This may limit the generalizability of the findings, particularly with regard to potential sex-specific differences in Long COVID symptoms and experiences.

PEM frequency was collected in a free-text field, as there is currently no standardized or validated measure to assess PEM frequency. While this approach limits comparability across studies and may have led to data entry errors, it also allowed participants to describe their individual experiences more precisely and without constraining response options. Furthermore, our analyses focused on a subgroup of individuals with Long COVID who experienced PEM, representing a subset of the broader Long COVID population. Consequently, their reported HRQoL may differ from those without PEM.

Another limitation is the use of the EQ-5D-3L, which - although well-established and widely used - may be less sensitive to subtle differences in health status compared to the newer EQ-5D-5L version [22]. Finally, the absence of longitudinal follow-up prevents conclusions about how temporal changes in PEM or fatigue may influence HRQoL over time.

Despite these limitations, this study contributes to a growing understanding of the association between PEM frequency, fatigue, and quality of life in individuals with Long COVID.

### Implications & future directions

Future research should incorporate standardized PEM assessment tools, objective physiological measures, and longitudinal designs to better capture symptom trajectories and their impact on HRQoL. Interventions targeting fatigue reduction and PEM management (e.g., pacing strategies, symptom-guided rehabilitation) may hold potential to improve HRQoL in Long COVID. Exploring additional moderators (e.g., comorbidities, psychological resilience) may help in tailoring individualized therapies.

In sum, this study reinforces that both PEM frequency and PEM severity are meaningful predictors of diminished HRQoL in Long COVID. Addressing them both is likely critical for optimizing recovery and patient care.

## Supplementary Information

Below is the link to the electronic supplementary material.

Supplementary Material 1

## Data Availability

The data are not publicly available. The data are available from the authors upon reasonable request.

## References

[1] World Health Organization. A clinical case definition of post COVID-19 condition by a Delphi consensus, 6 October 2021. 2021. https://www.who.int/publications/i/item/WHO-2019-nCoV-Post_COVID-19_condition-Clinical_case_definition-2021.1. Accessed 26 Sept 2025.

[2] CDC. Long COVID Signs and Symptoms. Long COVID. 2025. https://www.cdc.gov/long-covid/signs-symptoms/index.html. Accessed 26 Sept 2025.

[3] NICE. Overview | Myalgic encephalomyelitis (or encephalopathy)/chronic fatigue syndrome: diagnosis and management | Guidance | NICE. 2021. https://www.nice.org.uk/guidance/ng206?utm_source=chatgpt.com. Accessed 26 Sept 2025. 35438859

[4] Komaroff AL, Lipkin WI. Insights from myalgic encephalomyelitis/chronic fatigue syndrome may help unravel the pathogenesis of postacute COVID-19 syndrome. Trends Mol Med. 2021;27:895–906. 10.1016/j.molmed.2021.06.002. 34175230 10.1016/j.molmed.2021.06.002 PMC8180841

[5] Schmachtenberg T, Müller F, Kranz J, Dragaqina A, Wegener G, Königs G, et al. How do long COVID patients perceive their current life situation and occupational perspective? Results of a qualitative interview study in Germany. Front Public Health. 2023;11:1155193. 10.3389/fpubh.2023.1155193. 36969629 10.3389/fpubh.2023.1155193 PMC10034079

[6] Koczulla AR, Ankermann T, Behrends U, Berlit P, Berner R, Böing S, et al. S1-Leitlinie Long-/Post-COVID. Pneumologie. 2022;76:855–907. 10.1055/a-1946-3230. 36479679 10.1055/a-1946-3230

[7] Gardner E, Lockrey A, Stoesser KL, Leiser JP, Brown J, Kiraly B, et al. Challenges in Receiving Care for Long COVID: A Qualitative Interview Study Among Primary Care Patients About Expectations and Experiences. Ann Fam Med. 2024. 10.1370/afm.3145. 39191462 10.1370/afm.3145 PMC11419714

[8] Ottiger M, Poppele I, Sperling N, Schlesinger T, Müller K. Work ability and return-to-work of patients with post-COVID-19: a systematic review and meta-analysis. BMC Public Health. 2024;24:1811. 10.1186/s12889-024-19328-6. 38973011 10.1186/s12889-024-19328-6 PMC11229229

[9] Kerksieck P, Ballouz T, Haile SR, Schumacher C, Lacy J, Domenghino A, et al. Post COVID-19 condition, work ability and occupational changes in a population-based cohort. Lancet Reg Health Eur. 2023;31:100671. 10.1016/j.lanepe.2023.100671. 37366496 10.1016/j.lanepe.2023.100671 PMC10287546

[10] Vieth K, Hummers E, Roder S, Müller F, Wegener GS, Müllenmeister C, et al. How do people with long COVID cope with their symptoms and everyday limitations? A qualitative study with four focus groups. Z Für Evidenz Fortbild Qual Im Gesundheitswesen. 2025;195:68–77. 10.1016/j.zefq.2025.03.005. 10.1016/j.zefq.2025.03.005 40251050

[11] Abucar EAM, Kern M, Kurth T, Meierkord A, Gertler M, Seybold J, et al. Health-related quality of life up to 2 years after SARS-CoV-2 infection: a descriptive cohort study. Epidemiol Infect. 2025;153:e60. 10.1017/S0950268825000366. 40160155 10.1017/S0950268825000366 PMC12001138

[12] Scott ES, Lubetkin EI, Janssen MF, Yfantopolous J, Bonsel GJ, Haagsma JA. Cross-sectional and longitudinal comparison of health-related quality of life and mental well-being between persons with and without post COVID-19 condition. Front Epidemiol. 2023;3. 10.3389/fepid.2023.1144162. 10.3389/fepid.2023.1144162 PMC10910898 38455931

[13] BuSaad MA, Aldhawyan AF, Alattas BA, AlAlloush RS, Alharbi MA, Alkaltham NK, et al. Impact of Long COVID on Health-Related Quality of Life Among COVID-19 Survivors in Saudi Arabia. Healthcare. 2025;13:890. 10.3390/healthcare13080890. 40281839 10.3390/healthcare13080890 PMC12026523

[14] Schröder D, Heinemann S, Heesen G, Hummers E, Schmachtenberg T, Dopfer-Jablonka A, et al. Association of long COVID with health-related Quality of Life and Social Participation in Germany: Finding from an online-based cross-sectional survey. Heliyon. 2024;10. 10.1016/j.heliyon.2024.e26130. 10.1016/j.heliyon.2024.e26130 PMC10877341 38380019

[15] National Academies of Sciences E, Division H and M, Services B on HC, Administration C on the L-THES from C-19 and I for the SS, Spicer CM, Chu BX, et al. Selected Long-Term Health Effects Stemming from COVID-19 and Functional Implications. In: Long-Term Health Effects of COVID-19: Disability and Function Following SARS-CoV-2 Infection. National Academies Press (US); 2024. 39312610

[16] Mikuteit M, Heinemann S, Roder S, Niewolik J, Schröder D, Vahldiek K, et al. Long-term Consequences of COVID-19 and the Pandemic: Protocol for a Web-Based, Longitudinal Observational Study (DEFEAT). JMIR Res Protoc. 2022;11:e38718. 10.2196/38718. 36108134 10.2196/38718 PMC9611102

[17] von Elm E, Altman DG, Egger M, Pocock SJ, Gøtzsche PC, Vandenbroucke JP, et al. The Strengthening the Reporting of Observational Studies in Epidemiology (STROBE) statement: guidelines for reporting observational studies. Lancet Lond Engl. 2007;370:1453–7. 10.1016/S0140-6736(07)61602-X. 10.1016/S0140-6736(07)61602-X 18064739

[18] Devlin N, Parkin D, Janssen B. Methods for Analysing and Reporting EQ-5D Data. Cham: Springer International Publishing; 2020. 10.1007/978-3-030-47622-9. 33347096

[19] Greiner W, Claes C, Busschbach JJV, Graf von der Schulenburg J-M. Validating the EQ-5D with time trade off for the German population. Eur J Health Econ. 2005;6:124–30. 10.1007/s10198-004-0264-z. 19787848 10.1007/s10198-004-0264-z

[20] Marten O, Greiner W. Feasibility properties of the EQ-5D-3L and 5L in the general population: evidence from the GP Patient Survey on the impact of age. Health Econ Rev. 2022;12:28. 10.1186/s13561-022-00374-y. 35593942 10.1186/s13561-022-00374-y PMC9121571

[21] Brussoni M, Kruse S, Walker K. Validity and reliability of the EQ-5D-3L^™^ among a paediatric injury population. Health Qual Life Outcomes. 2013;11:157. 10.1186/1477-7525-11-157. 24044624 10.1186/1477-7525-11-157 PMC3848489

[22] Buchholz I, Janssen MF, Kohlmann T, Feng Y-S. A Systematic Review of Studies Comparing the Measurement Properties of the Three-Level and Five-Level Versions of the EQ-5D. PharmacoEconomics. 2018;36:645–61. 10.1007/s40273-018-0642-5. 29572719 10.1007/s40273-018-0642-5 PMC5954044

[23] Michielsen HJ, De Vries J, Van Heck GL. Psychometric qualities of a brief self-rated fatigue measure: The Fatigue Assessment Scale. J Psychosom Res. 2003;54:345–52. 10.1016/S0022-3999(02)00392-6. 12670612 10.1016/s0022-3999(02)00392-6

[24] Ordonez Cruickshank A, Poethko-Müller C, Schaffrath Rosario A, Sarganas G, Scheidt-Nave C, Schlack R. Prevalence of adults with fatigue in Germany: results of the ‘German Health Update 2023’ study. Eur J Public Health. 2024;34:Supplement_3:ckae144.2112. 10.1093/eurpub/ckae144.2112.

[25] Hinz A, Fleischer M, Brähler E, Wirtz H, Bosse-Henck A. Fatigue in patients with sarcoidosis, compared with the general population. Gen Hosp Psychiatry. 2011;33:462–8. 10.1016/j.genhosppsych.2011.05.009. 21749844 10.1016/j.genhosppsych.2011.05.009

[26] Universitätsklinikum Regensburg. Selbsttest zur Einstufung der Ermüdung (FAS). 2025. https://www.postlongcovid.de/newpage. Accessed 26 Sept 2025.

[27] de Vries J, Michielsen H, Van Heck GL, Drent M. Measuring fatigue in sarcoidosis: The Fatigue Assessment Scale (FAS). Br J Health Psychol. 2004;9:279–91. 10.1348/1359107041557048. 15296678 10.1348/1359107041557048

[28] Goodman E, Adler NE, Kawachi I, Frazier AL, Huang B, Colditz GA. Adolescents’ Perceptions of Social Status: Development and Evaluation of a New Indicator. Pediatrics. 2001;108:e31. 10.1542/peds.108.2.e31. 11483841 10.1542/peds.108.2.e31

[29] Demakakos P, Nazroo J, Breeze E, Marmot M. Socioeconomic status and health: the role of subjective social status. Soc Sci Med 1982. 2008;67:330–40. 10.1016/j.socscimed.2008.03.038. 10.1016/j.socscimed.2008.03.038 PMC2547480 18440111

[30] Morton F, Nijjar JS. Equation 5d: Methods for Analysing“EQ-5D”Data and Calculating“EQ-5D”Index Scores. 2025.

[31] Wickham H, François R, Henry L, Müller K, Vaughan D, Software P, et al. dplyr: A Grammar of Data Manipulation. 2023.

[32] Wickham H, Vaughan D, Girlich M, Ushey K, Software P, PBC. tidyr: Tidy Messy Data. 2024.

[33] Wickham H, Chang W, Henry L, Pedersen TL, Takahashi K, Wilke C, et al. ggplot2: Create Elegant Data Visualisations Using the Grammar of Graphics. 2025.

[34] Kassambara A. ggpubr: “ggplot2” Based Publication Ready Plots. 2025.

[35] Lüdecke D, Bartel A, Schwemmer C, Powell C, Djalovski A, Titz J. sjPlot: Data Visualization for Statistics in Social Science. 2025.

[36] Zhu [autH, cre, Travison T, Tsai T, Beasley W, Xie Y, et al. kableExtra: Construct Complex Table with kable and Pipe Syntax. 2024.

[37] Sarma A, Vogt A, Andrew A, Zvoleff A, knitr: A General-Purpose Package for Dynamic Report Generation in R. 2025. https://yihui.org.

[38] magrittr). SMB (Original author and creator of, Wickham H, Henry L, Software P, PBC [cph, fnd. magrittr: A Forward-Pipe Operator for R. 2025.

[39] Kassambara A. rstatix: Pipe-Friendly Framework for Basic Statistical Tests. 2025.

[40] Carlile O, Briggs A, Henderson AD, Butler-Cole BFC, Tazare J, Tomlinson LA, et al. Impact of long COVID on health-related quality-of-life: an OpenSAFELY population cohort study using patient-reported outcome measures (OpenPROMPT). Lancet Reg Health Eur. 2024;40:100908. 10.1016/j.lanepe.2024.100908. 38689605 10.1016/j.lanepe.2024.100908 PMC11059448

[41] Twomey R, DeMars J, Franklin K, Culos-Reed SN, Weatherald J, Wrightson JG. Chronic Fatigue and Postexertional Malaise in People Living With Long COVID: An Observational Study. Phys Ther. 2022;102:pzac005. 10.1093/ptj/pzac005. 35079817 10.1093/ptj/pzac005 PMC9383197

[42] Sun C, Liu Z, Li S, Wang Y, Liu G. Impact of Long COVID on Health-Related Quality of Life Among Patients After Acute COVID-19 Infection: A Cross-Sectional Study. Inq J Med Care Organ Provis Financ. 2024;61:00469580241246461. 10.1177/00469580241246461. 10.1177/00469580241246461 PMC11036910 38646896

[43] Vergouwe M, Birnie E, van Veelen S, Biemond JJ, Appelman B, Peters-Sengers H, et al. A Longitudinal Description of the Health-Related Quality of Life Among Individuals at High Risk After SARS-CoV-2 Infection: A Dutch Multicenter Observational Cohort Study. Open Forum Infect Dis. 2025;12:ofaf055. 10.1093/ofid/ofaf055. 39974282 10.1093/ofid/ofaf055 PMC11837172

[44] Stussman B, Camarillo N, McCrossin G, Stockman M, Norato G, Vetter CS, et al. Post-exertional malaise in Long COVID: subjective reporting versus objective assessment. Front Neurol. 2025;16:1534352. 10.3389/fneur.2025.1534352. 40337174 10.3389/fneur.2025.1534352 PMC12055772

[45] Tak CR. The health impact of long COVID: a cross-sectional examination of health-related quality of life, disability, and health status among individuals with self-reported post-acute sequelae of SARS CoV-2 infection at various points of recovery. J Patient-Rep Outcomes. 2023;7:31. 10.1186/s41687-023-00572-0. 36943643 10.1186/s41687-023-00572-0 PMC10029785

[46] Moens M, Duarte RV, De Smedt A, Putman K, Callens J, Billot M, et al. Health-related quality of life in persons post-COVID-19 infection in comparison to normative controls and chronic pain patients. Front Public Health. 2022;10:991572. 10.3389/fpubh.2022.991572. 36339175 10.3389/fpubh.2022.991572 PMC9632164

[47] Hvidberg MF, Petersen KD, Davidsen M, Witt Udsen F, Frølich A, Ehlers L, et al. Catalog of EQ-5D-3L health-related quality-of-life scores for 199 chronic conditions and health risks in Denmark. MDM Policy Pract. 2023;8. 10.1177/23814683231159023. 10.1177/23814683231159023 PMC10088414 37056295

[48] Di Tanna GL, Urbich M, Wirtz HS, Potrata B, Heisen M, Bennison C, et al. Health state utilities of patients with heart failure: a systematic literature review. Pharmaco Economics. 2021;39:211–29. 10.1007/s40273-020-00984-6. 10.1007/s40273-020-00984-6 PMC7867520 33251572

[49] Barz A, Berger J, Speicher M, Morsch A, Wanjek M, Rissland J, et al. Effects of a symptom-titrated exercise program on fatigue and quality of life in people with post-COVID condition – a randomized controlled trial. Sci Rep. 2024;14:30511. 10.1038/s41598-024-82584-4. 10.1038/s41598-024-82584-4 PMC11649701 39681609

