# Supplementary material for: Impact of post-exertional malaise frequency and fatigue in Long COVID patients on health-related quality of life
Source: Health Qual Life Outcomes. 2026 May 11;24:64. doi: 10.1186/s12955-026-02523-x (PMC13162394; doi:10.1186/s12955-026-02523-x)
Supplement: Supplementary file 1 — Supplementary Material 1 [file 12955_2026_2523_MOESM1_ESM.docx]

# Supplement

# S1. PEM-Frequency classification (open text fields)

## Daily

- täglich
- mehrmals täglich
- täglich bei leichter Anstrengung
- täglich wenn ich über körperliche Anstrengung meine Grenzen überschreite. Aber auch bei Konzentration: Gesprächen, Ausfüllen von Formularen, lesen.
- Täglich
- jeden Tag
- Wenn ich nicht aufpasse täglich

## Weekly

- Anfangs immer, aktuell seltener, ca 1xWoche
- Fast jeden 2. Tag
- Fast täglich
- Das hängt von vielen Faktoren ab. Leichte PEM aktuell fast täglich.
- fast täglich
- Mehrmals wöchentlich bis fast täglich
- Zur Zeit wöchentlich
- Mehrfach wöchentlich
- 3 - 4 wöchig
- wöchentlich
- Wöchentlich
- Nach jeder größeren Anstrengung, also 1-3× pro Woche durch Pendeln, weitere Strecken Gassi gegen oder zuviel Physiotherapie
- 5/woche
- 1-2 x pro Woche
- mehrmals pro Woche
- mindestens einmal die Woche, je nach Pacing
- Mehrmals wöchentlich
- durch konsequentes Pacen ca. 1 mal pro Woche
- 1xWoche
- 1 bis 2 mal wöchentlich
- Mehrmals pro Woche
- zur Zeit 2 - 3x pro Woche
- Mehrmals die Woche bis manchmal täglich - je nach körperlicher oder auch emotionaler Anstrengung
- 3 x wöchentlich
- 1 mal pro Woche
- mindestens zwei mal die Woche
- mehrmals wöchentlich, da bereits mein extrem reduzierter Alltag überfordernd ist (und ich wiederholt keinen Pflegegrad zugeteilt bekommen habe)
- Ca. 2-4 mal die Woche
- mehrmals wöchentlich
- Anfangs sehr häufig und regelmäßig, jetzt durch Pacing-Strategien etc. weniger, vielleicht ca. 1-3x pro Woche.
- 2-3 / Woche
- Wöchentlich, in Abhängigkeit von (nicht) erfolgreichen Pacing
- ca 2-3x/ Woche
- mehrfach in der woche
- Sehr unterschiedlich, 1 - 3 mal in dere Woche
- Mehrmals die Woche bei keinem strengend Pacing
- 4-5 pro Woche
- 2-3 mal die Woche
- 3-6× pro Monat
- 2-3x wöchentlich
- 1-2x/Woche je nach Pacing und Terminen
- 3x Woche
- 2x pro woche
- 4 -5 mal im Monat
- Ca. 1-3 mal pro Woche
- Jede Woche
- 4x pro Woche aber ohne Bettlägerigkeit
- 2x pro Woche
- 3-4 x pro Woche
- 6 pro Monat
- 2-3 pro Woche
- Ca. 1 x pro Woche
- Mehrmals in der Woche
- Inzwischen nur noch ca 1 mal pro Woche
- 1 mal pro Woche
- 2 bis 3 Mal in der Woche
- Minimal 2 mal pro Woche
- 3-4 die Woche
- Ca. 1-2 mal wöchentlich
- Anfang der Erkrankung (2022) sehr oft incl dauerhaften Zustandsverschlimmerung... ca 2-3x/ Woche
- alle 1-2 Wochen (Crash + PEM)
- 4 x im Monat
- 4-5x pro Woche
- 4 mal im monat
- 1 bis 2 mal die Woche
- kurze PEM (wenige Std.) mehrmals die Woche, längeranhaltende PEM ... ca. 2-3 x /Monat
- 4-5x im Monat
- 4-8x im Monat circa
- schwere PEM ca. einmal im Monat, leichte PEM wöchentlich
- 1-2 wöchentlich

## Monthly

- 1-4 mal im Monat
- fast wöchentlich, da meine aktuelle Arbeitsbeduíngung kein Pacing mehr zulassen
- Mehrmals im Monat
- 1-2x pro Monat
- Deutlich reduziert, aktuell nur noch körperlicher Aktivität, ca. 1 Monat
- 1-2x monatlich
- Je nach Häufigkeit der Überlastung, etwa 3-6 Mal pro Monat
- alle paar Wochen
- Alle 2 Wochen
- 1-2 mal pro Monat
- 2-4 mal monatlich
- alle ein bis zwei Wochen
- alle 2-3 Wochen
- Ca. 2-3x im Monat
- mehrmals pro Monat
- Mittlerweile nur noch ca.1x im Monat
- 1-2mal pro Monat
- Ca alle 4 Wochen
- Zweimal im Monat
- 2-3 mal pro Monat
- 1-4 Monat
- 1x im Monat
- 2x bis 4x im Monat
- 1-2 pro Monat, durch gutes Pacing
- 1 mal im Monat
- ca. 1-2x pro Monat
- ca. 2 Mal im Monat
- Ein bis zwei Mal im Monat
- einmal im Monat mindestens
- Monatlich
- Je nach Anstrengung, mind. 1x in zwei Wochen
- Heute selten, 1 bis 2x im Monat, wenn ich kein gutes Belastungsmanagement mache
- 1xalle2-3 Wochen
- alle 1-2 Monate
- 1 bis 2 mal im Monat, ich pace
- mindestens 1x im Monat
- Zu Beginn meiner Erkrankung vor 4 Jahren täglich... Ich habe PEM ca. 3-4 mal im Monat
- Regelmäßig (dank Pacing alle 7-10 Tage)
- Ich habe gelernt damit umzugehen, 1x im Monat
- 1/Monat
- Crashes 2 bis 3 Mal im Jahr. PEM mit spürbarer Verschlechterung von Symptomen monatlich.
- Mehrmals pro Monat
- Unregelmäßig im Durchschnitt 1x pro Monat
- 1x/Monat
- Derzeit nur noch alle 2 Wochen
- 1x Pro Monat
- 2-3 mal monatlich
- ca. 2 mal im Monat
- Unterschiedliche, mehrmals im Monat

## Less frequent

- Alle 2 Monaten
- Zu Beginn alle zwei bis drei Wochen, mittlerweile ungefähr alle 8-10 Wochen
- Alle 3 Monate
- Ca 3-4x / Jahr
- Weniger als ein Mal pro Monat
- alle 1-2 Monate

# S2. List of assessed comorbities

1. Hypertension
2. Heart failure
3. Coronary heart disease
4. Atrial fibrillation/flutter
5. Other cardiac arrhythmias
6. Type 1 diabetes
7. Type 2 diabetes
8. Asthma
9. Chronic obstructive pulmonary disease
10. Renal insufficiency / kidney failure
11. Gout
12. Gallstone disease
13. Severe obesity
14. Crohn’s disease / Ulcerative colitis
15. Chronic hepatitis
16. HIV
17. Psoriasis
18. Chronic wounds
19. Allergies
20. Atopic dermatitis
21. Thyroid disease
22. Rheumatoid diseases
23. Polymyalgia rheumatica
24. Other autoimmune diseases
25. Chronic pain
26. Migraine
27. Epilepsy / seizure disorders
28. Parkinson’s disease
29. Dementia
30. Schizophrenia / mania
31. Depression
32. Peripheral artery disease (PAD)
33. Other diseases

# S3. Descriptive statistics for fatigue severity and PEM frequency

| Variable | Median (Q1-Q3) | Mean ± SD |
| --- | --- | --- |
| Fatigue Severity | 28 (23-31) | 26.8 ± 6.48 |
| PEM Frequency | 3 (2-3) | 2.73 ± 0.71 |

# S4. Comparison of demographic and clinical characteristics between included participants and Long COVID participants with missing data

| **Variable** | **Included (n = 161)** | **Excluded (n = 377)** | **p-value** | **Notes** |
| --- | --- | --- | --- | --- |
| Age (mean ± SD) | 45.89 ± 10.94 | 48.39 ± 12.51 | **0.025** | Welch two-sample t-test |
| Gender | Female = 137 (85.1) | Female = 247 (65.5) | **0.157** | Pearson's Chi-squared |
|  | Male = 23 (14.3) | Male = 68 (18.0) |  |  |
|  | Diverse = 1 (0.6) | Diverse = 3 (0.8) |  |  |
|  | missing = 0 | missing = 59 (15.6) |  |  |
| Number of Comorbidities | Median = 3  IQR =2.5 | Median = 2  IQR = 2 | 0.259 | Wilcoxon rank-sum test; no significant difference |

# S5. EQ-5D domain profile by PEM frequency

| Domain/PEM-Frequency | Weekly (n=84) | Monthly (n=53) | Daily (n=19) | Less (n=5) |
| --- | --- | --- | --- | --- |
| Mobility | 66.7% (56) | 43.4% (23) | 78.9% (15) | 20.0% (1) |
| Self-care | 35.7% (30) | 20.8% (11) | 26.3% (5) | 20.0% (1) |
| Usual activities | 98.8% (83) | 81.1% (43) | 100.0% (19) | 60.0% (3) |
| Pain/discomfort | 95.2% (80) | 88.7% (47) | 100.0% (19) | 80.0% (4) |
| Anxiety/depression | 35.7% (30) | 26.4% (14) | 47.4% (9) | 20.0% (1) |

*EQ-5D-3L domain problems (levels 2–3) by PEM frequency, shown as % (n). Values indicate the proportion of participants reporting any problems (level 2 or 3) in each EQ-5D domain.*
